# Development of Acoustic Emission Sensor Optimized for Partial Discharge Monitoring in Power Transformers

**DOI:** 10.3390/s19081865

**Published:** 2019-04-18

**Authors:** Wojciech Sikorski

**Affiliations:** Institute of Electrical Power Engineering, Poznan University of Technology, Piotrowo 3A, 60-965 Poznan, Poland; wojciech.sikorski@put.poznan.pl

**Keywords:** acoustic emission (AE), ultrasonic transducer, partial discharge (PD), surface discharge, interturn discharge, oil–paper insulation, power transformer diagnostics, KLM model

## Abstract

The acoustic emission (AE) technique is one of the unconventional methods of partial discharges (PD) detection. It plays a particularly important role in oil-filled power transformers diagnostics because it enables the detection and online monitoring of PDs as well as localization of their sources. The performance of this technique highly depends on measurement system configuration but mostly on the type of applied AE sensor. The paper presents, in detail, the design and manufacturing stages of an ultrasensitive AE sensor optimized for partial discharge detection in power transformers. The design assumptions were formulated based on extensive laboratory research, which allowed for the identification of dominant acoustic frequencies emitted by partial discharges in oil–paper insulation. The Krimholtz–Leedom–Matthaei (KLM) model was used to iteratively find optimal material and geometric properties of the main structures of the prototype AE sensor. It has two sensing elements with opposite polarization direction and different heights. The fully differential design allowed to obtain the desired properties of the transducer, i.e., a two-resonant (68 kHz and 90 kHz) and wide (30–100 kHz) frequency response curve, high peak sensitivity (−61.1 dB ref. V/µbar), and low noise. The laboratory tests confirmed that the prototype transducer is characterized by ultrahigh sensitivity of partial discharge detection. Compared to commonly used commercial AE sensors, the average amplitude of PD pulses registered with the prototype sensor was a minimum of 5.2 dB higher, and a maximum of 19.8 dB higher.

## 1. Introduction

Power transformers are a strategic component of the electric power system. It is commonly believed that the oil–paper insulation system is designed in order to work reliably for 30–40 years. However, there are many power transformers installed all around the world, which operate without failure above this age. The asset management strategy developed by most power distribution companies, which is directed towards an extension of transformer life, results mostly from the high cost of the device purchased as well as long delivery time, reaching over a year. However, such policy increases the probability of catastrophic failure occurrence, which is followed by a number of negative consequences, such as a threat to the life of the substation staff, significant economic losses, environmental hazards (oil leaks or fire), and local blackouts [1,2,3,4,5]. Therefore, recently, an increase of requirements towards the reliability of the exploited power transformers and the demand for professional services and diagnostics solutions has been observed. Such a strategy extends the time of safe exploitation of older units [6,7,8,9,10]. While analyzing cases of power transformer breakdowns described in the literature or reports of power distribution companies, one may notice that many of them are related to accelerated degradation of the insulation system, which might have been caused by the high activity of partial discharges [1,11,12,13,14]. For that reason, monitoring of PD intensity and the dynamics of its changes may be an important indicator to predict impending failure. The effectiveness of PD detection depends not only on the applied measurement technique, but also on the configuration of the measurement system, particularly on PD sensors performance. The highest sensitivity of PD pulse detection may be obtained using a measurement setup that complies with the IEC 60270 standard (so-called conventional electrical method) [15,16]. Under ideal measuring conditions (shielded high-voltage laboratory), the conventional electrical method allows detection of low-energy PD pulses (apparent charge < 0.1 pC). Unfortunately, PD detection in field conditions, due to the high level of electromagnetic disturbances (switching operations and corona discharge in transmission lines), is practically impossible [17]. Mainly for that reason, online monitoring systems available on the market or currently developed by research centers are based only on unconventional PD detection methods. This group includes dissolved gas analysis (DGA) in oil [18,19,20], detection of electromagnetic waves in the high-frequency (HF) [21,22] and ultrahigh frequency (UHF) ranges [23,24,25,26,27], and the acoustic emission method (AE), which, apart from detection and on-line monitoring of PD, also locates PD sources [28,29,30,31,32]. It should be underlined that sensitivity of these techniques highly depends on sensor position in a power transformer tank and its distance from the PD source as well as on the construction and parameters of the sensor [33].

In the acoustic emission method, mostly piezoelectric contact transducers are used for PD detection. It should be noted that in the last few years the research works on alternative solutions have been conducted. Sikorski in [34] presented an active dielectric window (ADW). It is a new concept of combined acoustic emission (AE) and electromagnetic PD detector intended for assembly in the transformer’s inspection hatch. A similar approach was presented by Siegel et al. [35], but, in this case, a sensor able to detect AE and UHF signals was mounted through an oil drain valve. Due to the high cost of commercial AE sensors, some researchers propose low-budget solutions, based on, among others, the application of piezoelectric diaphragms (buzzers) [36].

Leading manufacturers, like Physical Acoustics or Vallen Systeme, offer several dozen different types of piezoelectric sensors, which cover the demand of the majority of research fields and diagnostic techniques based on the acoustic emission phenomenon. Depending on application type and, resulting from it, the frequencies of ultrasonic signals registered, transducers are divided into three categories: low frequency (20–100 kHz), standard frequency (100–400 kHz), and high frequency (>400 kHz) sensors. The category of standard frequency sensors is the most numerous. They are, among others, used for drying process monitoring of wood, hot reheat pipe crack detection, or integrity testing of pressure vessels, metallic, and composite structures. Low-frequency AE sensors are usually recommended for structural health monitoring of large concrete and geologic structures, leakage detection in liquid pipelines, and corrosion screening of flat bottom storage tanks. In turn, high-frequency sensors are applied in AE testing of small specimens [37,38]. Wideband AE sensors with a flat frequency response curve (at the cost of less sensitivity) are a separate category. Despite lower efficiency, this type of transducers is ideal in cases where the frequency of AE signals generated by the investigated phenomenon is unknown or when acquired AE signals are broadband by nature.

The recommendations of the leading manufacturers regarding the optimal choice of sensor for PD detection in oil-filled transformers are far less unambiguous than in case of the remaining AE applications. Vallen Systeme suggests the use of a transducer operating in the range between 100 kHz and 400 kHz or low-frequency transducer (20–100 kHz) when noise is low. From the variety of the offered several dozens of sensors, the producer selected two models for PD detection, i.e., VS30 (20–80 kHz) and VS75 (30–120 kHz, frequency peak 75 kHz) [38]. In turn, Physical Acoustics offered five models of transducers (D9241A, R6D, R15D, R30D, and R50D), for which monitoring of large power transformers is specified as one of the typical applications [39]. Providing such sensors is quite confusing since they work in different frequency ranges. For instance, the operating frequency range for sensor type D9241A is from 20 kHz to 60 kHz (with frequency peak 30 kHz) and for sensor type R15D from 50 kHz to 400 kHz (resonant frequencies: 75 kHz and 150 kHz), whereas for sensor type R50D between 100 kHz and 700 kHz (resonant frequencies: 100 kHz and 500 kHz) [39]. 

The presented information show that, so far, the manufacturers have not elaborated an AE sensor specially designed and optimized for PD detection in oil-filled power transformers. It is hard to blame only the producers because there are at least several reasons for such a situation. First, power transformer diagnostics using the AE method are still being run on a fairly small scale, particularly compared to such industrial applications of the AE method such as structural health monitoring of large concrete and steel structures, fatigue and fracture materials research, bearing condition monitoring, or leakage detection in pipelines. Second, scientists investigating AE signals emitted by PD—depending on the type of the applied ultrasonic transducer and measurement set-up configuration—draw quite different conclusions (see details in Section 2). Third, recommendations included in official documents of international organizations establishing standards (IEC, IEEE, and CIGRE) are very general. In the Guide for the Detection of Acoustic Emissions from Partial Discharges in Oil-Immersed Power Transformers issued by IEEE in 2000 [40], the AE sensor for PD detection is defined as a piezoelectric displacement transducer operating in its compression mode and has a resonant frequency (for longitudinal waves) in the 120 to 160 kHz range. Users are recommended to apply a band-pass filter (with lower and upper cutoff frequencies of 100 kHz and 300 kHz) to eliminate most of the signals that are not associated with PDs, e.g., vibrations caused by the magnetostrictive action of the core (Barkhausen noise), oil pumps, and cooling fans. Simultaneously, it was noted that depending on location and type of PD source, some group of users find that a low-frequency AE sensor (e.g., 60 kHz) is more useful, particularly when higher-frequency signals are attenuated. However, the IEEE guide criticizes this type of sensor for being more susceptible to external or other mechanical signals.

In the revised version of the IEEE C57.127 guide issued seven years later, more attention was paid to the problem of the adequate sensor selection [41]. First of all, it was highlighted that the main frequency of low-energy PDs (e.g., partial discharge in oil and PD in voids or gas bubbles) is 100 kHz and for larger discharges (surface discharges, turn-to-turn discharges, and arcing) frequencies should decrease (20–100 kHz). Furthermore, the low-frequency AE waves are less attenuated. For that reason, part of the users—particularly for factory and laboratory applications—choose sensors with resonant frequencies for longitudinal waves of 60 kHz. However, fearing of external disturbances and noises occurring in the band between 20 kHz and even 60 kHz, most users apply sensors with the resonant frequency of 150 kHz. Unfortunately, this choice is not related to the partial discharges frequency at all. Rather, this is due to the fact that 150 kHz standard transducers are easy available on the market (e.g., Physical Acoustics R15α and R15D, Vallen VS150, Soundwel SR150, Bangos AES150, Acoustic Emission Consulting i150, Fuji Ceramics AE154DL, AE144S, etc.). The 150 kHz standard AE sensor may reduce the noise level compared to the 60 kHz resonant frequency sensor, but it will also strongly reduce PD detection sensitivity [41,42].

In 2009 CIGRE Working Group D1.33 published a technical brochure titled Guidelines for Unconventional Partial Discharge Measurements [43], in which one may find information that PD occurrence is a source of acoustic emission signals of frequencies in a band between 10 kHz and 300 kHz. In turn, in a brochure issued in 2015 titled Guidelines for Partial Discharge Detection using Conventional (IEC 60270) and Unconventional Methods [44], according to the CIGRÉ Working Group D1.37 experts, the frequency band of the AE signals emitted by PDs is definitely wider and is in the range from 20 to even 1000 kHz. The change in recommendations might have been caused by the fact that part of the researchers prefers broadband multiresonant transducers.

In the standard IEC TS 62478: High voltage test techniques—Measurement of partial discharges by electromagnetic and acoustic methods published in 2016 by the International Electrotechnical Commission (IEC)—the detailed guidelines regarding AE sensors were not included [45]. The document informs that for PD detection, typically the ultrasonic frequency range is employed (approximately 20 kHz to 250 kHz), as well as the audible range (approximately 100 Hz to 20 kHz). It additionally underlines that the frequency ranges used for acoustic detection are chosen depending on the insulation system (solid, liquid, or gaseous), for which the AE method is being employed.

The newest document, in which the issue of AE sensor selection has been raised, is a brochure Partial Discharges in Transformers elaborated by CIGRÉ Working Group D1.29 and issued in February 2017 [46]. This document suggests using transducers working in a band of 10 to 300 kHz, where in order to obtain the highest PD detection sensitivity, resonant type piezoelectric sensors with resonance frequency between 60 kHz and 150 kHz are recommended.

The relatively low PD detection sensitivity of the AE method used in power transformers diagnostics is considered as one of its main disadvantages. This results directly from the physical nature of the PD phenomenon because only a small part of the energy of a PD current pulse is transformed into mechanical energy in the form of acoustic waves. Another reason is the AE wave attenuation—the combined effect of scattering, absorption, and reflection of sound waves, which occurs on the wave propagation path between PD source and the ultrasonic transducer. Therefore, the use of a proper ultrasonic transducer is so important from the AE method efficiency point of view [33].

The above-presented state of affairs has become a motivation to design and manufacture an AE sensor optimized for detection and monitoring of PD in oil-filled power transformers. As a result of the conducted research, a prototype ultrasonic transducer was developed, which sensitivity of partial discharges detection is higher than in the case of the most popular commercial AE sensors. In addition, its most important advantage is its very simple structure and low production costs. The prototype sensor presented in this article may find application in the AE systems specially designed for locating or online monitoring of partial discharges in large power transformers. In the theoretical part of the manuscript (Section 2), the results of the previous works on acoustic frequencies emitted by partial discharges in oil–paper insulation were discussed. The investigated types of PDs and electrode systems used in the experiment were described in Section 3. Parameters of the measurement setup, in which the frequencies of AE signals generated by partial discharges, were identified were presented in Section 4. Basing on the obtained measurement results (Section 5), a prototype AE sensor was designed and manufactured. The details concerning this task were discussed in Section 6. Finally, the prototype sensor was tested, in order to evaluate its performance compared to commonly used commercial AE sensors (Section 7). 

## 2. Acoustic Frequencies Emitted by Partial Discharges in Oil–Paper Insulation—Previous Works

One of the first papers dealing with the frequency analysis of AE signals emitted by partial discharges in oil–paper insulation was presented by R.T. Harrold in 1975 [47]. Ultrasonic signals were recorded using a wideband transducer, which was composed of a lithium sulfate disc (0.147 cm thick, 2.28 cm diameter) with stainless-steel electrodes. In his research, the author limited the analyzed frequency band to the range from 150 kHz to 1.75 MHz. On the basis of the obtained results, the following conclusions were formulated. (1) Ultrasonic frequency spectra from 150 kHz to 1.75 MHz emitted by different types of PD in oil had not revealed any unique frequency of high acoustic energy; (2) using resonant transducers in the band above 150 kHz, AE from various PD sources should easily be detected, but signals received at the lower frequencies were higher and the frequency spectrum of a point-to-pressboard plane source in oil might have cut off near 300 kHz; and (3) detection of PD sources using acoustic emission technique was more readily achieved using resonant transducers operating in the 20 kHz to 100 kHz range. 

In another of Harrold’s works [48], the acoustic emission signal generated by sparks and arcs in mineral oil was investigated. Partial discharge pulses were recorded with both narrowband and wideband ultrasonic transducers: Brüel & Kjaer type 4135 condenser microphone (10 Hz–100 kHz), Brüel & Kjaer type 4131 condenser microphone (20 Hz–20 kHz), Celesco type LC-10 hydrophone (1 kHz–100 kHz), self-produced wideband hydrophone (1 kHz–2 MHz), and Physical Acoustics Corporation contact microphone, type 15-12 (up to 500 kHz). Studies showed that (as previously theoretically predicted), high energy (kJ) arcs radiate maximum acoustic levels in the low frequency region from ~120 Hz to ~10 kHz, whereas, low energy (µJ) PD (microsparks) have maximum acoustic emission at higher frequencies in the ~10 kHz to ~400 kHz range.

Howells and Norton [49] were one of the first to publish a comprehensive report, in which they assessed the possibility of practical application of the AE method for diagnostics of power transformers. The authors performed laboratory tests on three small distribution 10 kVA transformers with deliberately modeled defects to generate and register AE pulses from partial discharges. The applied AE measurement system consisted of a piezoelectric resonant-type transducer (Dunegan S140 L/D with 140 kHz nominal resonant frequency) and 100 kHz high-pass filter. Such configuration strongly influenced the frequency parameters of the registered AE pulses. Their dominant frequency (~150 kHz) practically coincided with the resonance frequency of the ultrasonic transducer. The authors also presented the results obtained during the induced voltage tests with PD measurement of two large power transformers (230 kV/56 MVA and 230 kV/200 MVA). The same type of ultrasonic transducer was used to detect AE pulses, but this time the high-pass filter with a lower cutoff frequency (20 kHz instead of 100 kHz) was used. For both tested transformers, AE pulses were recorded only at higher voltage levels (130% and 150% rated voltage, respectively). The frequency-domain analysis showed that most of the AE signal energy from PD is transmitted in the low-frequency band (from 20 kHz to about 80 kHz), and the rest in the range of 140 to 170 kHz, that is close to the resonant frequency of the transducer.

In the work of Zhu et al. [50], the relations between various types of PD in the transformer and their frequency spectra of AE were studied. For this purpose, different electrode systems were used, in which insulation defects were modeled: (1) PD of protrusions in transformer; (2) PD of point electrode with different curvature; (3) PD along barrier surface in transformer; (4) PD in gaseous void in solid insulation of transformer; and (5) PD in the thick paper insulation of high voltage connection of transformer. Furthermore, the authors investigated the frequency of acoustic signals generated by magnetic noises. In this study, two kinds of AE sensor with piezoelectric elements were used. One was wideband and the other narrowband. Their maximum frequency response was 700 kHz and 200 kHz, respectively. Based on the obtained results, the following conclusions were drawn. (1) Obtained peak frequencies in the frequency spectra of AE from PD distributed within 70 to 150 kHz; (2) frequency spectra analysis showed that frequencies of Barkhaussen noise were lower than 20 kHz and frequencies of magneto-mechanical AE were within 20–65 kHz; (3) to minimize the negative impact of magnetic noises, frequency bandwidth of 70–180 kHz was recommended for the AE measuring system of PD detection in power transformer; and (4) various types of PD emit signals of different frequencies, which made it possible to identify insulation defects.

Bengtsson et al. [51] performed a transducer independent determination of the acoustic frequencies emitted by a partial discharge in oil. For this purpose, the experiment was based on the phenomenon of single-slit diffraction, and the registration of AE pulses was performed using two 100 kHz hydrophones. The first one was a reference and the second one was mounted in a computer controlled moving frame. It was found that the main signal content from low-energy PDs ~100 pC magnitude is ~100 kHz (91 kHz and 99 kHz are peak frequencies). In the later publication [42], the authors stated that due to the short AE signal duration, the frequency distribution was rather wide. However, due to the effect that high-frequency components are strongly attenuated by pressboard, they prefer acoustic sensors in the 20 to 120 kHz range, which is lower than recommended by users, who are afraid of core noises. The authors also presented interesting results of the experiment, in which PDs in the insulation of a paper-wound conductor were recorded simultaneously using low and high-frequency sensors. This experiment shows that the detection sensitivity for this type of PD signals is higher using low-frequency sensors (peak frequency was ~50 kHz) as the high-frequency components are severely attenuated.

In another Bengtsson paper [52], for recording PD pulses in transformer oil, the UAEA 250 transducer with two resonant frequencies—125 kHz and 250 kHz—was used. In the point-to-plate electrode system immersed in oil, the main frequency of registered AE signals overlapped with the first resonance frequency of the UAEA transducer (125 kHz). In turn, in the case of creeping discharges, most of the energy of the AE signal was transmitted in the low-frequency range from approximately 25 to 40 kHz and close to the center frequency of the transducer (110–130 kHz).

In research carried out by Sakoda et al. [53], ultrasonic waves caused by a single PD pulse in oil were measured. The results of these measurements were analyzed by means of an FFT in order to understand the properties of the elastic waves and distinguish the kinds of detectable elastic waves. Broadband ultrasonic transducer (1000 kHz) was used in the research. The results presented in the paper proved that almost all energy of AE pulses from PD in oil was transmitted in the lower frequency band (up to about 100 kHz) and the dominant frequency was about 25 kHz.

In turn, Boczar [54] analyzed the AE signals emitted by (1) surface discharges in oil, (2) gas bubble discharges in oil, and (3) discharges in indeterminate potential particles moving in oil. Detection of AE pulses was performed by a wideband (from 10 kHz to 1 MHz) piezoelectric transducer type 8312 of Brüel & Kjaer, with a standard 40 dB built-in preamplifier. The creeping discharges generated low-frequency AE signals (below 100 kHz), with most of the energy being carried in the band from about 70 to 90 kHz. The other two types of PD generated signals in a much wider frequency band.

Kundu et al. [55] studied PD on conductor protrusions in the transformer and PD along barrier surface in the transformer. Point-to-plane electrode systems were used to model the PD on protrusions, plane-to-plane electrodes with pressboard insulation and rod-to-plane electrodes with pressboard insulation were used to model surface discharge. The resonance-type 150 kHz AE sensor was used to acquire PD signals. For the PD in oil, the average peak frequency was 128 kHz and the median frequency was 138 kHz. AE signals from surface discharges had definitely lower frequencies. In a plane-to-plane electrode system, the average peak frequency of AE was 60 kHz and in rod-to-plane electrode system configuration was 50 kHz. The average of median frequency was 78 kHz and 88 kHz, respectively. 

In the work of Sikorski [56], most of the basic types of partial discharges (ten types in total) occurring in oil–paper insulation were examined. Based on the results of the research, the following conclusions were formulated. (1) Each of the investigated PD forms generates repeatable and unique AE signals, (2) results of frequency-domain analysis strongly depend on the type of the applied sensor, (3) the low-frequency (30 kHz) sensor is more sensitive in detection of high-energy PD like surface discharges than wideband sensor, (4) high-energy creeping sparks generate AE signal in the 20 to 40 kHz frequency band, and (5) low-energy PD, like discharges in gas bubbles or in internal gas voids, emit short AE pulses in the higher frequency band (100–300 kHz). The conclusions from the model tests were confirmed by measurements carried out on power transformers.

## 3. Investigated Types of Partial Discharges and Electrode Systems Used in Experiment

Basing on the literature [57,58,59], surface and interturn discharges were selected for the research, in relation to their high energy and strongly destructive character for power transformer insulation system. Additionally, PDs in oil were investigated, which usually are the initial form of the surface and interturn discharges.

### 3.1. Surface Discharges

Various terms for surface discharges, such as sliding discharge, creeping discharge, or surface tracking discharge, can be found in the literature [60,61]. Surface discharge is the most dangerous type of PD that can lead to a catastrophic failure of a power transformer under normal operating conditions [62]. This phenomenon occurs in the oil/pressboard barrier system, which is the weakest and most problematic area in the power transformer insulation system, because of the accumulation of water molecules (due to moisture migration), contaminations (iron or copper particles, corrosive sulfur) and other aging products (gaseous and solid hydrocarbons). Generally, all insulation systems of the transformer can be treated as systems of the oil barrier type in which the initiation and development of PDs always take place in oil [59]. In an ideal setup of this type, the electric field is perpendicular to the barrier surface and parallel (tangential) to the spacing elements (strips and spacers). In this configuration, the ratio of electric field strength in the barrier to electric field strength in the oil channels is inversely proportional to the ratio of the relative permittivity of the barrier (ε = 3.6–4.7) and oil (ε = 2.2). Thus, the electric field strength in the barriers does not exceed 60–70% of the electric field strength in the oil channels. A breakdown of barriers that are located perpendicularly to the force lines of the electric field can only be a result of discharges in oil. Spacers in oil ducts in the ideal oil barrier system are ‘unbreakable’ because the cross-stress here is almost the same as stress in the oil channels. The cross-strength of the spacing elements is always greater than their surface dielectric strength. Therefore, the development of PD on strips or spacers can be caused almost exclusively by a loss of surface dielectric strength [63]. The electrical stress on the oil/pressboard interface contains both tangential and normal components of the electric field. However, the formation of surface discharge over the pressboard barrier is more assisted by the tangential component and, according to Sokolov, progresses in four stages [64]:

Stage 1. The discharge begins in the oil, on the edge of the outer winding coil (small area with very strong electric field), and then develops in the form of plasma channel (leader), which is more or less consistent with the direction of electric field force lines. As a result, it comes to the breakdown of an oil duct, between the outer winding coil, and the nearest pressboard barrier.

Stage 2. Upon reaching the barrier, the plasma channel spreads on the barrier surface in the form of creeping sparks, which create—due to high temperature—permanent carbonized, partially conductive paths, so-called ‘black marks’. At the initial stage of surface discharge development, there are also ‘white marks’ observed that indicate a drying out process of the pressboard through moisture evaporation [60,65,66].

Stage 3. Electric field forces that accompany the surface discharges are capable of pulling water and oil out of the cellulose pores, which results in microscopic sparking within the pressboard. High moisture content and an inhomogeneous distribution in the pressboard barrier cause a dangerous current concentration on a narrow path. Then carbonized traces are generated even at very low currents, ~0.1 mA. Along with the development of this phenomenon, the electric conductivity of the path grows—until breakdown. The electric field forces can accumulate water molecules into the shortest path between the ‘electrodes’, facilitating the initiation of the high-energy, long creeping sparks [62]. 

**Figure 1:**
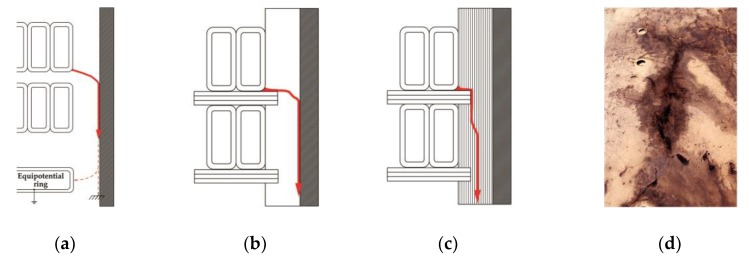
Possible scenarios of surface discharge formation in transformer insulation system: (**a**) flashover along the barrier to the earthed construction part or equipotential ring (the flashover bypasses the intercoil spacers); (**b**) flashover across the surface of intercoil spacer; the discharge may later develop along the lateral surface of the strip (or on the surface of barrier—as shown in Figure 1a; (**c**) breakdown of distance strip; and (**d**) photo of a fragment (~450 × 800 mm) of the pressboard barrier strongly degraded by surface discharges (numerous carbonized traces are visible).

Stage 4. High-energy sparking not only causes the formation of carbonized traces on the pressboard surface, but also leads—due to dissociation of oil molecules—to the formation of hydrogen [67], gaseous hydrocarbons (mainly C_2_H_2_ and CH_4_), and wax-like substances [68]. As the path length increases, the apparent charge of the surface discharges decreases to ~500 pC‒5 nC, and it is high enough to continue the degradation of cellulose. The permanent destruction of cellulose fibers occurs under the influence of PD with an apparent charge of ~1 nC. The development of surface discharge can continue from minutes to even years, until the treeing conductive path cause shunting of an essential part in the transformer insulation resulting in a powerful arc [69]. The possible scenarios of surface discharge formation in transformer insulation system are illustrated in Figure 1.

The main three causes of surface discharge initiation are as follows [59].

**Figure 2:**
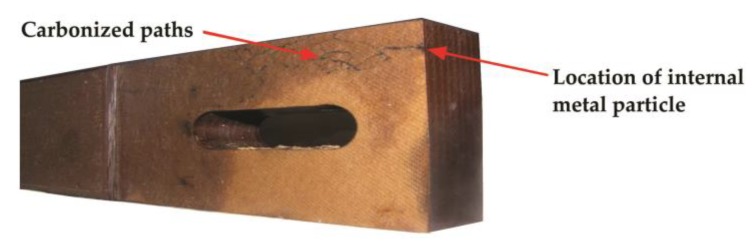
The location of an embedded metal particle under the surface of pressboard beam (the PD tracking originated from this area).

**Figure 3:**
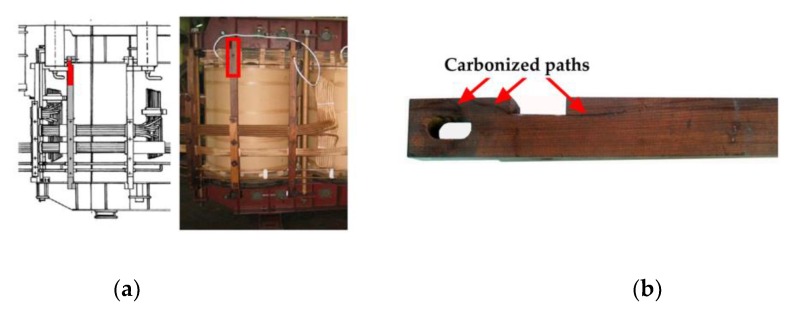
Example of surface discharge occurrence in a 16 MVA power transformer caused by too small of a distance between the supporting beam and the winding: (**a**) construction scheme and the picture of transformer active part with marked location of the defect and (**b**) picture of supporting beam with visible permanent carbonized paths.

1. The occurrence of sources of intense ionization, such as
  - air bubbles that can get into the transformer tank during the oil filling process [56],
  - free water molecules that have settled out of the insulation and oil into a separate layer,
  - emulsified water, which is supersaturated in solution but has not yet totally separated from the oil,
  - the violent release of water vapor bubbles from cellulose to oil (“bubble effect”) [70],
  - local electrical overstress in the oil wedge (at the oil–pressboard–electrode triple junction),
  - gas bubbles formed by the cavitation in transformer oil. The cavitation phenomenon (defined as the formation of discontinuity areas in a fluid) can be triggered by high temperature (hot spots), oil pumps or core/windings vibrations [71],
  - metal particle contamination [31] (see Figure 2).
2. The abnormal configuration of the insulation system, that stimulate the generation of surface discharges, such as
  - contact or too small distance between pressboard barrier and winding—this situation is usually caused by winding radial deformations (bucklings);
  - touch of pressboard barrier to bushing connection terminal; and
  - too small of a distance between the supporting beam and transformer tank wall or windings (see Figure 3) [29].
3. The occurrence of local electrical stresses large enough to initiate creeping discharges (tangential electrical component stress ≥1 kV/mm) [59].

As mentioned before, a necessary condition for the appearance of surface discharges is a presence of the tangential component of the electric field (perpendicular to the dielectric surface). Insulation systems, in which tangential components are primary phenomena (i.e., forced by electrode system), are divided into two groups: (1) insulation systems, which normal components are negligibly small and (2) insulation systems, in which they play an important role.

**Figure 4:**
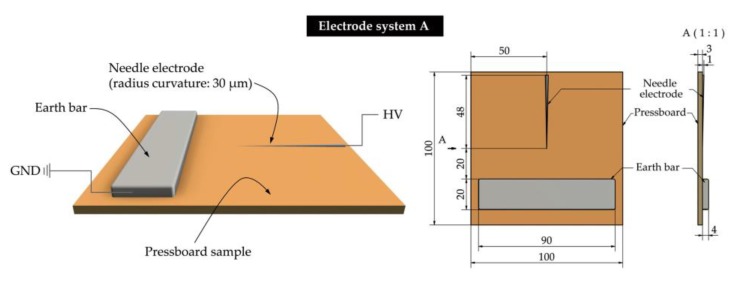
The applied electrode system for generation of surface discharges on pressboard sample in oil with a negligibly small normal component of electric field—electrode system A.

**Figure 5:**
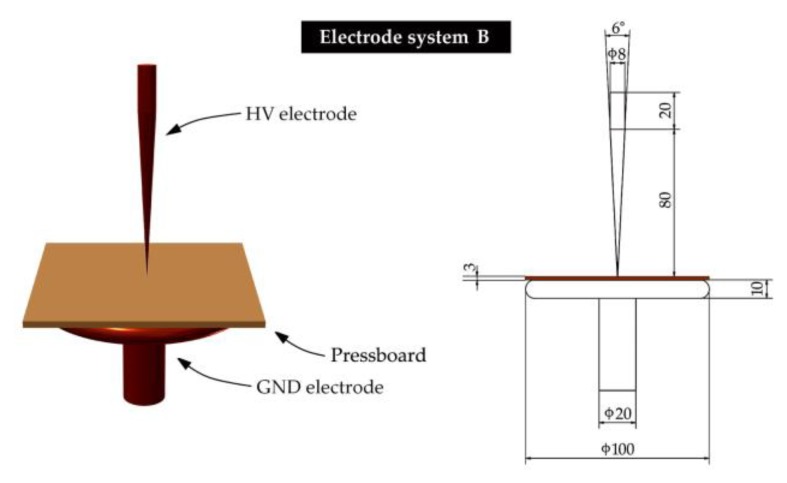
The applied electrode system for generation of surface discharges on a pressboard sample in oil with significant normal component of electric field—electrode system B.

The first group is typical for all spacing elements, e.g., pressboard strips spacing oil channels or pressboard spacers spacing coils. For generating surface discharges of this type, the presented in Figure 4 electrode system was used (hereinafter referred to as "electrode system A"). A typical representative of the second group (so-called Toepler systems) is an insulation system of bushing insulator or insulation between two windings of different heights (in this case at the end of a lower winding, there occur both tangential and normal components) [60,72,73,74]. This type of surface discharges was generated in the electrode system (hereinafter referred to as "electrode system B"), which was presented in Figure 5. 

### 3.2. Interturn Partial Discharges

Another very dangerous type of PD for power transformer insulation system is interturn (intercoil) discharge. The following gives the reasons for the occurrence of these discharges.

**Figure 6:**
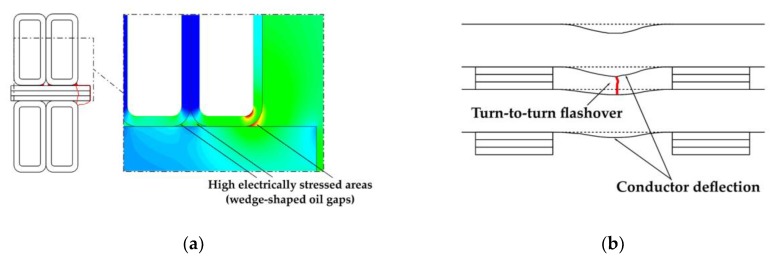
Possible reasons for interturn discharges in power transformer: (**a**) high electrically stress areas in wedge-shaped oil gaps and (**b**) too small insulation spacing caused by excessive axial deflection of conductors (coils).

1. High electrical stress in wedge-shaped oil gaps [75] (see Figure 6a),
2. axial deformation of windings (axial collapse by excessive axial compression, conductors tilting, axial bending of conductors and excessive deflection, winding insulation damage, and/or rupture) [58,59] (see Figure 6b),
3. excessive moisture content leading to a concentration of gas bubbles in oil gaps and ducts [70], and
4. paper and pressboard surface contamination with conductive particles and oil aging products [61].

It is estimated that interturn discharges, compared to surface discharges, in a significantly shorter time, may lead to power transformer catastrophic failure. During the defect development, the energy of discharges grows quickly from relatively low level approximately 500–1000 pC to over 100 nC, which causes insulation breakdown and turn-to-turn flashover. 

**Figure 7:**
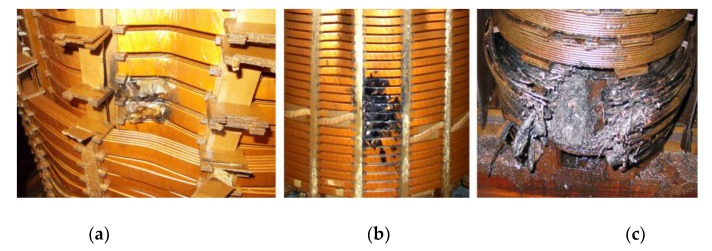
Pictures presenting consequences of destructive action of interturn discharges: (**a**) breakdown of paper insulation in the place of the deformed winding, (**b**) a vast area of carbonized winding insulation, (**c**) complete destruction of windings and insulation.

**Figure 8:**
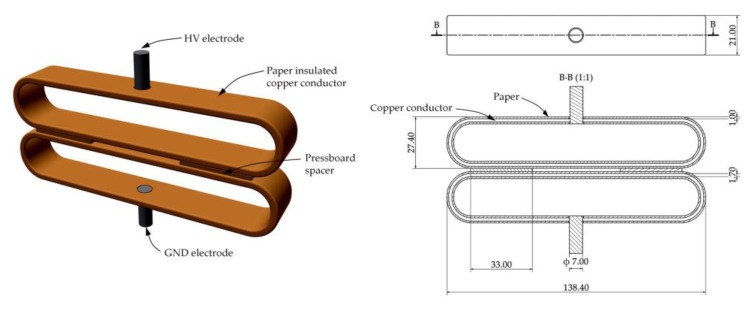
The applied electrode system for generation of interturn discharges.

The examples of the destructive influence of interturn discharges on power transformer insulation system were shown in Figure 7, whereas the electrode system, in which this type of discharges was generated, is shown in Figure 8. 

### 3.3. Partial Discharges in Oil

**Figure 9:**
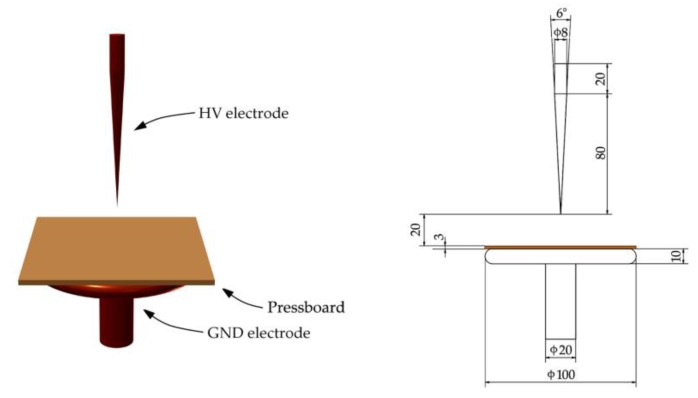
The applied electrode system for the generation of PD in oil.

For generating PD in oil, the needle–plate electrode system, shown in Figure 9, was applied. Of course, such an electrode system is unacceptable in the main insulation system of the power transformer. However, its properties give a view on the consequences of constructive or technological oversights, e.g., when needle electrode (screw or sharp edge) is a grounded constructive element. Additionally, one may assume that every kind of discharge in a transformer insulation system is always—in its initial stage of development—a discharge in oil, as plasma has no contact with electrodes. The interturn insulation may be an example in which most cases of breakdown occur at the edges of the coil and the discharge is initiated in the oil gap (see Figure 1a).

## 4. Measurement Setup

For investigating the frequency of acoustic signals emitted by partial discharges, broadband (10–1000 kHz) ultrasonic transducer type Olympus V101-RB of relatively flat frequency response curve (without resonance peaks) was used. For comparison, acquisition of AE pulses emitted by PDs was additionally conducted using three piezoelectric sensors, which are commonly applied in power transformer diagnostics, i.e., resonant standard frequency AE sensor type PAC R15D, low-frequency AE sensor type PAC D9241A, and wideband multiresonant AE sensor type PAC WD. The parameters of the transducers applied in the investigation are presented in Table 1.

**Figure 10:**
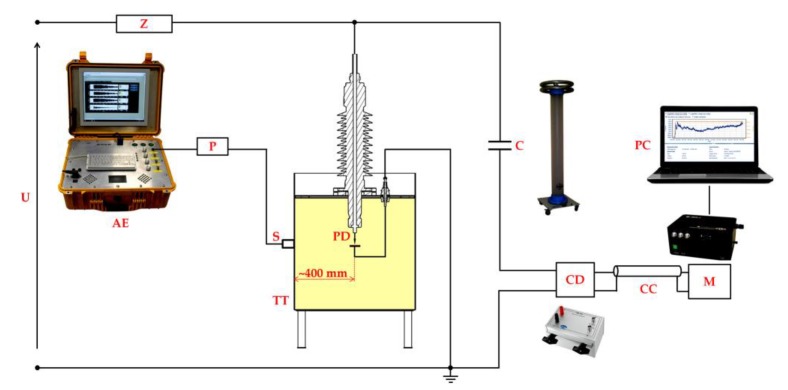
Schematic diagram of the measurement setup: U = high-voltage supply; Z = short-circuit current limiting resistor; AE = four-channel acoustic emission measuring system; P = preamplifier; S = acoustic emission sensor; TT = oil-filled transformer tank; PD = electrode system for partial discharge generation; C = coupling capacitor; CD = coupling device (measuring impedance); CC = connecting cable; M = conventional partial discharge measuring instrument; PC = computer.

The electrode systems for generation of the investigated forms of partial discharges described in Section 3 were put in an oil-filled transformer tank of dimensions 1200 × 800 × 750 mm. During the measurements, one of the four selected ultrasonic transducers was mounted exactly in front of the defect at distance ~400 mm. As an ultrasonic couplant, silicone grease was used. Acoustic signals emitted by PDs were registered using measurement system type PDtracker Portable I (Poznan University of Technology, Poznan, Poland) [76], equipped with four analog input channels (with simultaneous sampling at up to 20 MS/s) and preamplifiers with 40 dB gain and a 20–1000 kHz bandpass filter. Monitoring of partial discharge activity was performed with the use of conventional PD measuring instrument type Doble PD-Smart (Doble Engineering Co, Boston, MA, USA) and test circuit according to IEC 60270 standard (Figure 10) was used. AE signals were registered for such a value of test voltage, at which PD activity was stable (nonextinguishing). The electrical parameters of the investigated types of discharges are presented in Table 2.

## 5. Results and Discussions

For each of the investigated PD forms, 500 AE waveforms were registered. Then, for all collected pulses, the average power spectrum density (PSD) was determined based on the peak hold averaging technique. Peak hold averaging is performed at each frequency line separately, retaining peak levels from one FFT record to the next. Because peak hold averaging retains the peak levels of the averaged quantities, it is possible to detect all harmonic components that transfer most of the signal energy [77,78].

**Figure 11:**
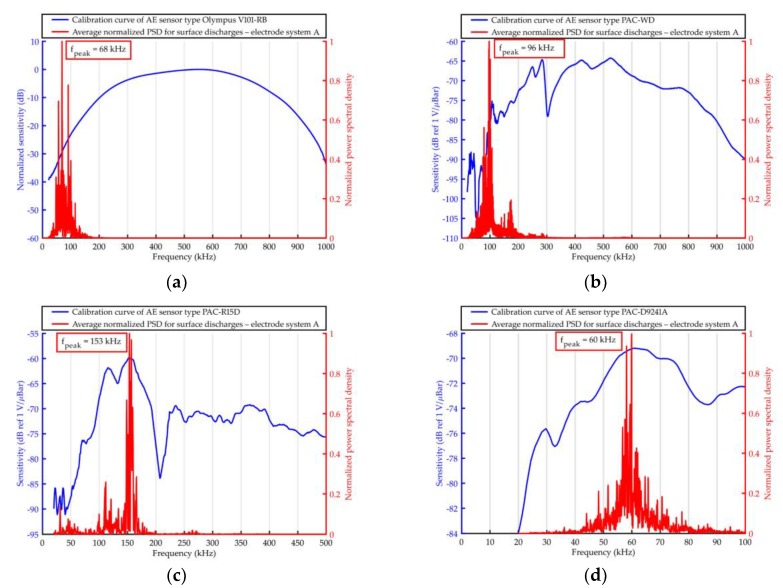
Average normalized power spectrum density (PSD) determined for acoustic emission (AE) signals emitted by surface discharges (generated in electrode system A) and registered with the use of ultrasonic sensor type: (**a**) Olympus V101-RB, (**b**) PAC WD, (**c**) PAC R15D, and (**d**) PAC D9241A.

**Figure 12:**
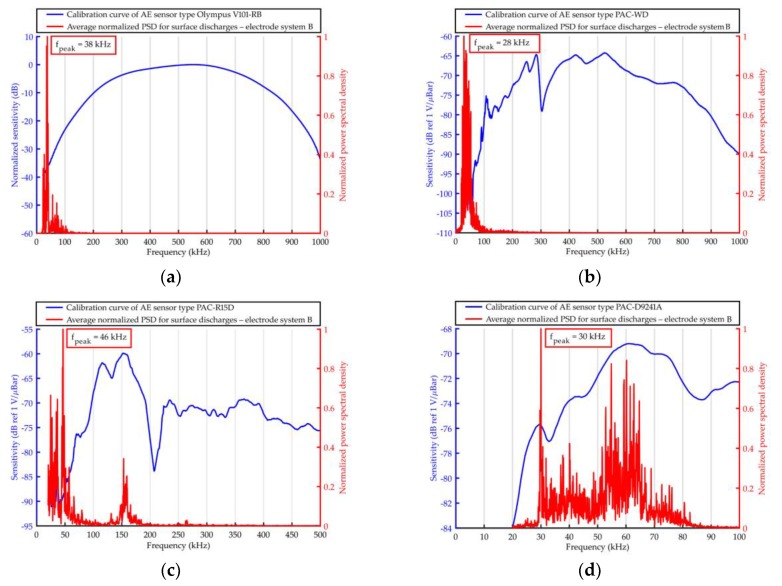
Average normalized PSD determined for AE signals emitted by surface discharges (generated in the electrode system B) and registered with the use of ultrasonic sensor type: (**a**) Olympus V101-RB, (**b**) PAC WD, (**c**) PAC R15D, and (**d**) PAC D9241A.

**Figure 13:**
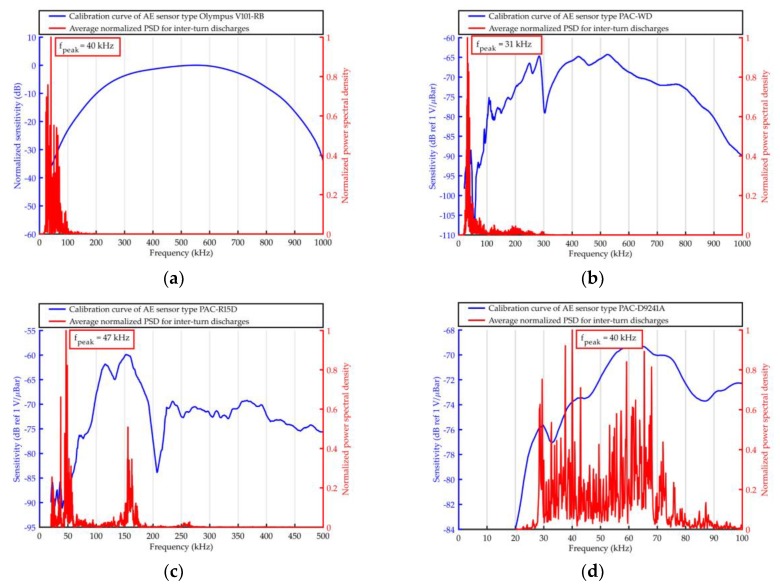
Average normalized PSD determined for AE signals emitted by interturn discharges and registered with the use of ultrasonic sensor type: (**a**) Olympus V101-RB, (**b**) PAC WD, (**c**) PAC R15D, and (**d**) PAC D9241A.

**Figure 14:**
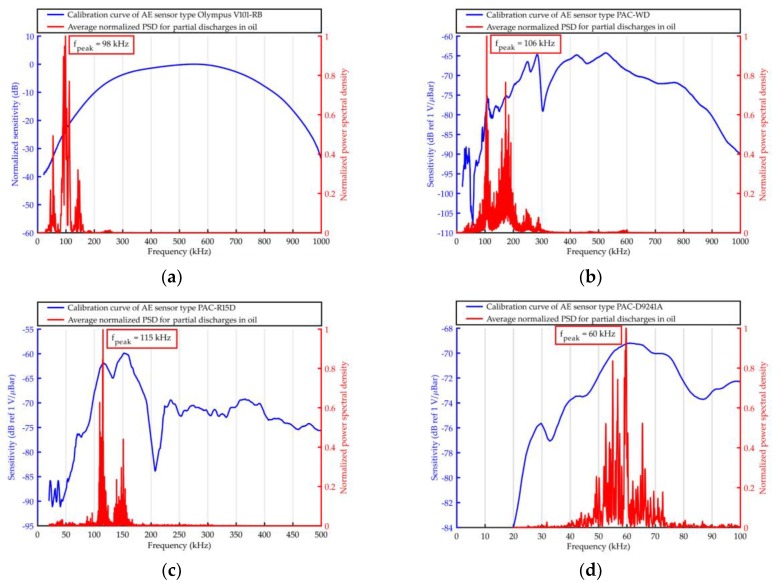
Average normalized PSD determined for AE signals emitted by partial discharges in oil and registered with the use of ultrasonic sensor type: (**a**) Olympus V101-RB, (**b**) PAC WD, (**c**) PAC R15D, and (**d**) PAC D9241A.

The elaborated averaged PSD were shown together with the frequency response curves of the used AE sensors in Figure 11, Figure 12, Figure 13 and Figure 14.

For the elaborated average PSD, besides peak frequency *f_peak_* (point in the spectrum at which the peak magnitude is observed), frequency centroid *f_centroid_* and weighted peak frequency 〈*f_peak_*〉 were determined as well (Table 3). Frequency centroid is an average weighted frequency that takes into account the whole spectrum (it indicates the location of the "center of mass" of the PSD). Thus the frequency centroid gives a fuller picture of the AE signal’s frequency content than the peak frequency. The parameter is described with the formula below.

(1) $$f_{centroid}=\frac{\sum_{k=1}^{N}k\cdot F\left[k\right]}{\sum_{k=1}^{N}F\left[k\right]},$$

where *F*[*k*] is the magnitude corresponding to bin *k* in the frequency spectrum. 

In turn, weighted peak frequency 〈*f_peak_*〉 is a geometric mean of frequency peak *f_peak_* and frequency centroid *f_centroid_*:

(2) $$\langle f_{peak}\rangle =\sqrt{f_{peak}\cdot f_{centroid}}$$

Based on the results obtained with the use of Olympus V101-RB, the following conclusions may be drawn.

- From all the investigated PD forms, AE signals of the lowest frequency were emitted by the surface discharges generated in the electrode system B, in which the normal component of the electric field plays a significant role. The frequency of the AE signals ranged between 20 kHz and 110 kHz, wherein 95% of the energy was transferred in a narrow band from 22 kHz to 42 kHz. In turn, surface discharges generated in electrode system A, in which normal component is negligibly small, emitted AE signals of higher frequencies. This kind of surface discharge transferred 95% of the acoustic wave energy in the band between 48 kHz and 100 kHz. Frequency centroid was 78.6 kHz and a peak frequency 68.3 kHz (for surface discharges generated in electrode system B, it was 44.5 kHz and 37.6 kHz, respectively).
- The values of frequency parameters of interturn discharges were slightly higher (by about 2–5 kHz) than parameters of surface discharges generated in electrode system B. The dominating frequency band, in which 95% of the AE pulses energy was transferred, was in the range of 20 to 68 kHz.
- AE signals of the higher frequency generated discharges in oil, which, opposite to the remaining PD forms, had broadband character. In PSD, one may distinguish three separate frequency bands, i.e., 43–64 kHz, 80–117 kHz, and 131–155 kHz. However, the largest part of the energy (90.3%) was transferred in the band of 80–117 kHz, and the peak frequency was equal to 98.1 kHz.

Considering the results obtained with the use of the remaining transducers, one may formulate the following additional conclusions.

**Figure 15:**
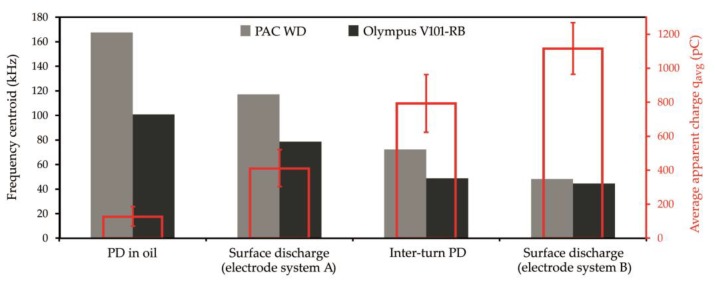
Influence of type and energy (value of apparent charge) of partial discharge on the centroid frequency of registered AE signal.

- Independently of the kind of the used transducers for surface discharges with significant normal components of the electric field (electrode system B) and for interturn discharges, AE signals of low frequency (<50 kHz) were registered.
- Resonant and multiresonant transducers do not enable to precisely detect frequencies of AE signal emitted by PD (they coincide with main or side-band resonance frequency).
- In the case of both broadband transducers (Olympus 101-RB, PAC WD), a clear relation between the investigated form of the partial discharge and frequency centroid of emitted AE signals was observed. In Figure 15, one may see that the more energetic the given type of the discharges was, the more “center of mass” of the spectrum (frequency centroid) moved to the lower frequencies.

**Figure 16:**
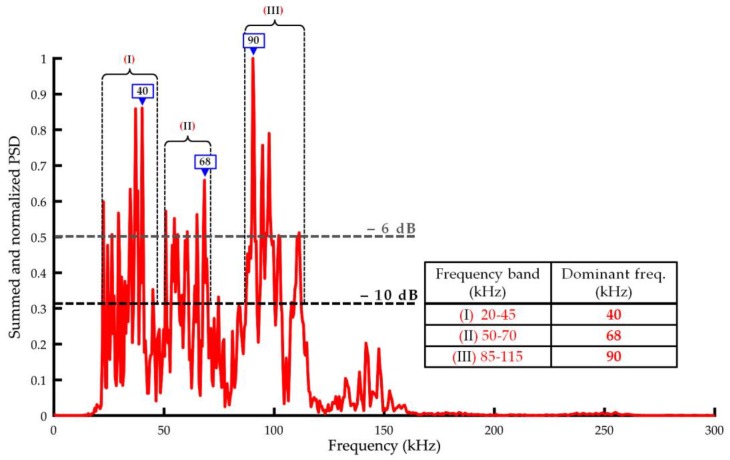
Summed and normalized PSD obtained for all investigated types of PD.

In order to obtain a more complex picture of the frequencies of the AE signals emitted by the PDs, all power density spectra collected using a broadband sensor type V101-RB (Figure 16) were summed and normalized. The analysis of the resultant spectrum allows to distinguish three main frequency bands, in which the most energy of acoustic waves originating from surface discharge, interturn discharge, and discharge in oil was transferred, i.e., 20–45 kHz, 50–70 kHz, and 85–115 kHz. Furthermore, in these bands, one may distinguish the dominating frequencies, which equal to 40 kHz, 68 kHz, and 90 kHz, respectively. Basing on this, one may formulate a thesis that the optimal AE sensor for PD detection in power transformers should be a multiresonant transducer, in which the calibration response curve has peaks at the frequencies mentioned above. One should not forget the possible sources of disturbances occurring in power transformer. Luckily, the majority of them, like pumps and fans, emit acoustic signals of frequency below 30 kHz. However, in extreme cases, the frequency of noise associated with the deformation of magnetic domains in the transformer core may even reach 50 kHz [41]. Nevertheless, usually acoustic signals generated by the Barkhausen effect have too low energy to excite the transducer. Additionally, they are effectively attenuated by the paper insulated windings and thick pressboard barriers. Therefore, one may assume that the ideal compromise linking high sensitivity of the PD detection and resistance to external disturbances is an ultrasonic transducer with a bandwidth of 30 to 115 kHz and resonances at 68 kHz and 90 kHz. In Section 6, each stage of designing and manufacturing of the transducer, which meets the above requirements, is presented. 

## 6. Design and Fabrication of Prototype AE Sensor

**Figure 17:**
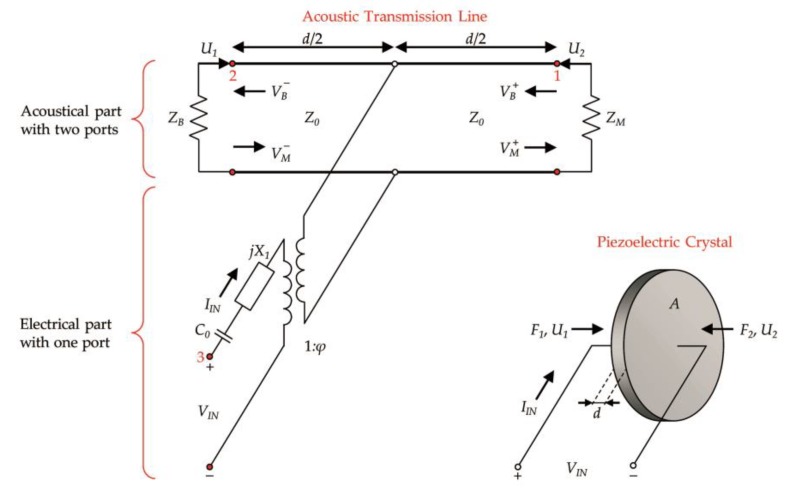
Schematic diagram of the Krimholtz–Leedom–Matthaei (KLM) model of a piezoelectric transducer.

For prediction of transducer resonant frequencies, the Krimholtz–Leedom–Matthaei (KLM) [79] model is currently most often used. Compared to other ultrasonic transducer models, like RLC [80], Mason [81] and Redwood model [82], its main advantage is the separation of the acoustical and electrical parts of the transduction process (Figure 17). 

Due to that, it is possible to analyze these parts individually to improve the design of all transducer components, i.e., matching layer, piezoelectric element, backing layer, and electrical matching network. Moreover, the KLM model helps to design and optimize the transducers of a more complex, multilayer structure [83,84]. A detailed description of the KLM model may be found in the source publication [79], as well as many other papers in which authors present the design process of piezoelectric transducers’ based on this method [85,86,87]. 

The input parameters consist of the thickness of the piezoelectric material *d*, the area of the electrodes *A*, and the acoustic impedances of the main transducer elements: the piezoelectric crystal *Z*_0_, backing layer *Z_B_*, and matching layer *Z_M_*. Moreover, the model comprises the input capacitance *C*_0_ and input electrical reactance *X*_1_. The acoustical side of the KLM model is fixed to the electrical side by a transformer with a turns ratio (1:*φ*). This transformer converts the electrical signal into the proper acoustic values.

The equations for input parameters of the KLM model are given below [34].

(3) $$Z_{0}=\rho cA=A\sqrt{\rho c_{33}^{D}}$$

(4) $$C_{0}=\frac{\varepsilon_{33}^{S}A}{d}$$

(5) $$X_{1}=\frac{h_{33}^{2}A}{\omega^{2}Z_{0}}\sin\left(\frac{\omega d}{c}\right)$$

(6) $$\varphi =\frac{\omega Z_{0}}{2h_{33}\sin(\omega d/2c)}$$

(7) $$h_{33}=k_{t}\sqrt{c_{33}^{D}/\varepsilon_{33}^{S}}$$

where *ρ* is the density of the piezoceramic material (kg/m^3^), *c* is the velocity of longitudinal acoustic waves (m/s), $c_{33}^{D}\;$is the elastic stiffened for constant electric displacement (N/m^2^), $\varepsilon_{33}^{S}\;$is the clamped (high-frequency) dielectric constant, *φ* is the electromechanical transformer ratio of the KLM model, $h_{33}$ is the piezoelectric constant (V/m), and $k_{t}$ is the electromechanical coupling factor.

Based on the value of the acoustic impedance (in Rayl or Pa·s/m^3^) of piezoelectric crystal *Z*_0_, the matching layer *Z_M_*, backing layer *Z_B_*, and the input impedances for both layers may be determined as follows

(8) $$Z_{IN,M}=Z_{0}\frac{Z_{M}+jZ_{0}\tan(\omega d/2c)}{Z_{0}+jZ_{M}\tan(\omega d/2c)}$$

(9) $$Z_{IN,B}=Z_{0}\frac{Z_{B}+jZ_{0}\tan(\omega d/2c)}{Z_{0}+jZ_{B}\tan(\omega d/2c)}$$

Considering the transmission line theory, the value of the input impedance *Z_IN_* of the electrical port can be calculated based on Equation (10):

(10) $$Z_{IN}=\frac{1}{j\omega C_{0}}+jX_{1}+\frac{Z_{a}}{\varphi^{2}}$$

where $Z_{a}\;$is the impedance as seen looking into the acoustic transmission line:

(11) $$Z_{a}=\frac{Z_{IN,M}Z_{IN,B}}{Z_{IN,M}+Z_{IN,B}}$$

*Z_IN_* relates to the real impedance measured by the impedance analyzer. The minimum impedance frequency on the *Z_IN_(f)* plot is the resonant frequency, at which the piezoceramic element vibrates most readily and most efficiently transforms the mechanical energy into electrical energy. In turn, the maximum impedance frequency is also the anti-resonance frequency. 

In order to ensure efficient transmission of acoustic waves energy from mineral oil through steel power transformer tank to the transducer’s piezoelectric components, it is necessary to apply matching layer, which optimal impedance value should be the geometric mean of the impedances of steel (*Z_s_*) and the piezoelectric material (*Z_PZT_*):

(12) $$Z_{M}=\sqrt{Z_{S}Z_{PZT}}$$

Assuming the acoustic impedance of the steel equals *Z_S_* = 46.2 MRayl and selected piezoelectric ceramics *Z_PZT_* = 31.5 MRayl (see Table 5), then the optimal value of matching layer acoustic impedance should total 38.15 MRayl. Therefore, high-density alumina (Al_2_O_3_), with an impedance that equals approx. 36‒37 MRayl, is usually the first choice material for the design and manufacture of matching layer for AE sensors, that are supposed to be mounted at all kinds of metal structures (transformers, reactors, storage tanks, pipelines, bridges, etc.). In the case of the designed sensor, the disc with the diameter of 25 mm and height of 1 mm manufactured from high-purity alumina and acoustic impedance equal to ~37.9 MRayl is the matching layer. Basic parameters of the ceramics are summarized in Table 4.

Two sensing elements (piezoceramic discs) with opposite polarization directions and different geometries were used as active components. The technology based on two piezoelectric elements is used by Physical Acoustics Company [39]. This way, for example, a multiresonant sensor type PAC WD is produced, in which the piezoceramic ring and the axially placed disc are the active elements. Such a fully differential design offers a number of advantages, of which the most important are

- multiresonance frequency response curve (it has two main resonance frequency in thickness vibration mode),
- high sensitivity and low noise (because differential preamplifier multiplies the difference between two input signals and simultaneously eliminates common-mode noise), and
- broader bandwidth in comparison to single-ended resonant AE sensor.

For manufacturing active elements, piezoelectric ceramics PZT type 5A (Navy Type II) was selected, which has high charge sensitivity and operates over a wide temperature range. Properties of the used piezoelectric material are summarized in Table 5.

**Figure 18:**
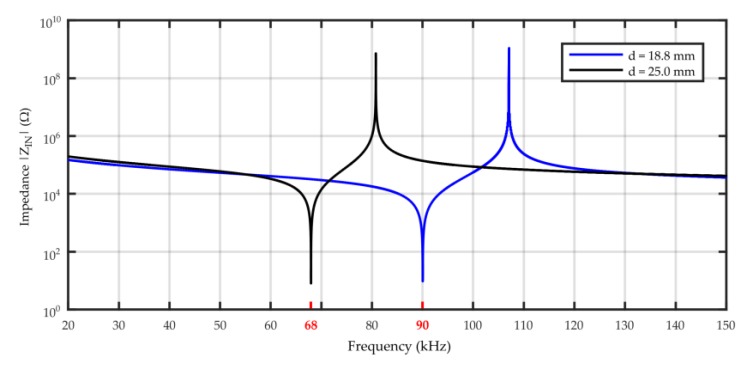
Simulated input impedance *Z_IN_(f)* measurement based on the KLM model for piezoelectric discs with a height of 18.8 mm and 25 mm.

Calculations based on the KLM model showed that the resonance frequencies of thickness vibration mode will be equal to 68 kHz and 90 kHz if piezoelectric discs of height 25 mm and 18.8 mm, respectively, are applied. The presented in Figure 18 results of simulation in form of input impedance characteristics *Z_IN_(f)* also allowed to recognize anti-resonance frequencies (at maximum impedance), which for selected PZT discs totaled 80.8 kHz and 107.1 kHz.

**Figure 19:**
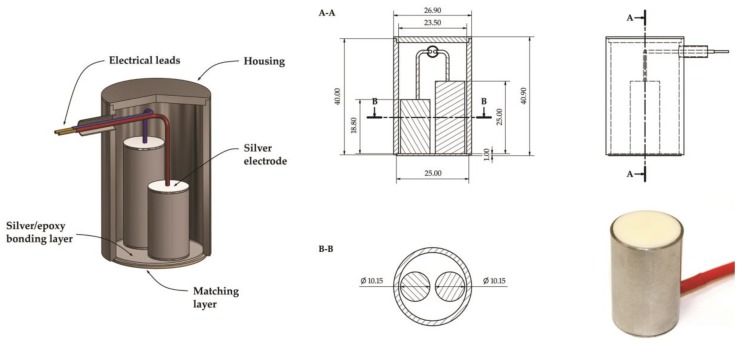
Schematic diagram, technical drawing with dimensions and photo of prototype AE sensor named A6890.

Thickness vibration mode is the main operating mode of AE sensor because partial discharges in oil generate only longitudinal acoustic waves. However, the transducer is mounted outside the power transformer tank. In practice, it means that besides longitudinal waves, it also registers shear waves (structure-borne waves), which propagate in power transformer tank wall. This proves that resonance frequencies of radial vibration mode should also be equal to 68 kHz and 90 kHz. A transducer with such properties would surely guarantee excellent sensitivity of PD detection. Unfortunately, the main limitation in its realization is the necessity of use of piezoelectric discs with very large diameters. In order to obtain the resonance frequency of radial vibration mode, the disc diameter should be equal to 22 mm, and even ~29 mm for 68 kHz. Such a transducer would have large dimensions and weight. Therefore, in the selection of 10.15 mm diameter of piezoelectric elements, mainly the matters of optimal use of matching layer surface and minimal weight of the transducer were taken into account. Piezoelectric elements were mounted to the matching layer using silver–epoxy electrically conductive adhesive and equipped in signal leads. Schematic diagram, technical drawing with the dimensions and picture of the prototype transducer were presented in Figure 19. The transducer was given a name A6890, which refers to its two main resonant frequencies: 68 kHz and 90 kHz.

**Figure 20:**
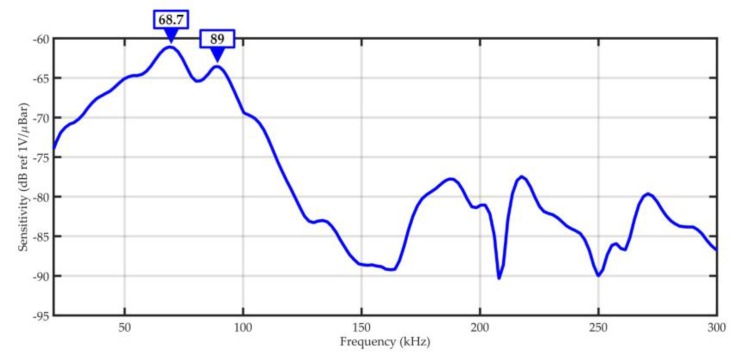
The frequency response curve of the prototype AE sensor type A6890.

The calibration of the prototype transducer was carried out according to ASTM E976-10: Standard Guide for Determining the Reproducibility of Acoustic Emission Sensor Response [88]. Analysis of the presented in Figure 20 frequency response curve shows that main resonant frequencies (68.7 kHz and 89 kHz) of the prototype transducer only slightly differ from the desired values, i.e., 68 kHz and 90 kHz. Moreover, the transducer is characterized by high sensitivity (peak sensitivity: −61.1 dB ref. V/µbar) and wide bandwidth (30–100 kHz).

**Figure 21:**
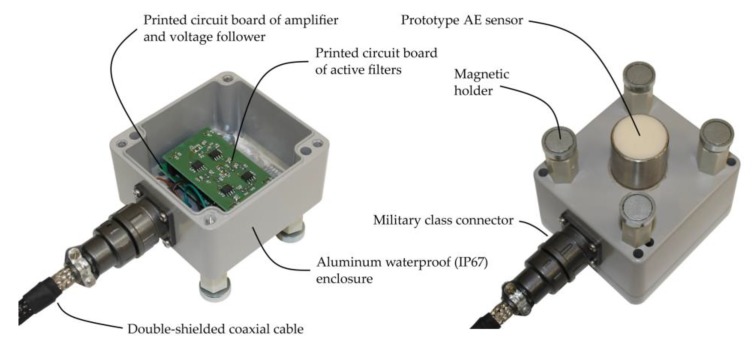
Prototype AE sensor integrated with IP67 waterproof preamplifier housing equipped with magnetic holders.

Moreover, in frames of the conducted research works, the AE transducer was manufactured in version dedicated to operate in online monitoring system. For that purpose, the AE sensor was integrated with a waterproof housing, in which printed circuit boards of a low-noise preamplifier with 40 dB gain and active filters were placed. Due to the use of low-noise instrumentation and operational amplifiers (AD8421BRZ, ADA4898, and AD813ARZ), the noise level at the output of this AE signal conditioning unit with 40 dB gain and with connected ultrasonic transducer did not exceed 1.6 mV_p-p_. The detailed information concerning these circuits was presented by the author in [34]. The housing was equipped with magnetic holders, which assure constant contact pressure between the transducer front face and transformer tank wall (Figure 21).

## 7. Sensitivity Tests of Prototype AE Sensor

**Figure 22:**
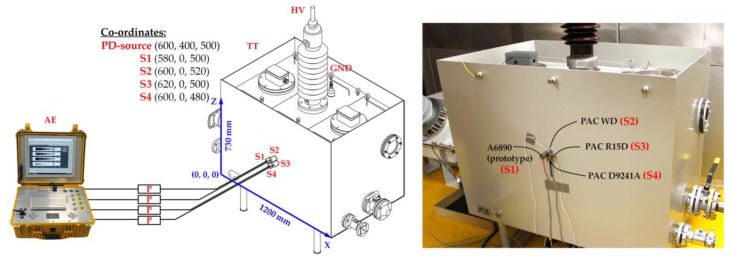
Schematic diagram of measurement setup and photo of the transformer tank installed in electromagnetic shielded laboratory: AE = four-channel acoustic emission measuring system; P = preamplifier with 40 dB gain; S1, S2, S3, and S4 = acoustic emission sensors; HV = high-voltage bushing; GND = ground; TT = oil-filled transformer tank.

In order to assess the efficiency of the prototype AE sensor, laboratory research was carried out, during which surface discharges, interturn discharges, and discharges in oil were generated. AE signals emitted by PDs were registered simultaneously with prototype transducer and three commercial ones, i.e., PAC R15D, PAC D9241A, and PAC WD. The ultrasonic transducers were located on power transformer tank in order to be placed closest to each other and at the same distance from the PD source equal to ~400.5 mm. The schematic diagram of the measurement setup is presented in Figure 22.

The detection sensitivity of the prototype PD sensor was assessed based on the comparative analysis with earlier mentioned three commercial transducers. In order to achieve this goal, for collected *N* = 500 sets of AE waveforms, the average signal amplitude gain was calculated as follows

(13) $$\bar{a_{v}}=\frac{\sum_{i=1}^{N}20\log\left(\frac{A_{p}}{A_{ref}}\right)}{N}$$

where $\bar{a_{v}}$—average signal amplitude gain (dB); *A_p_*—amplitude of AE signal registered with prototype sensor (V); *A_ref_*—amplitude of AE signal registered with one of commercial reference sensor (V); and *N*—the number of AE waveforms sets.

**Figure 23:**
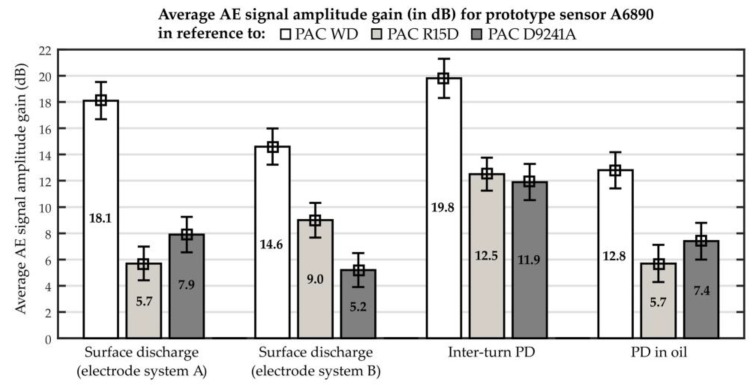
Average AE signal amplitude gain for prototype sensor A6890 in reference to commonly used commercial sensors: PAC WD, PAC R15D, and PAC D9241A.

**Figure 24:**
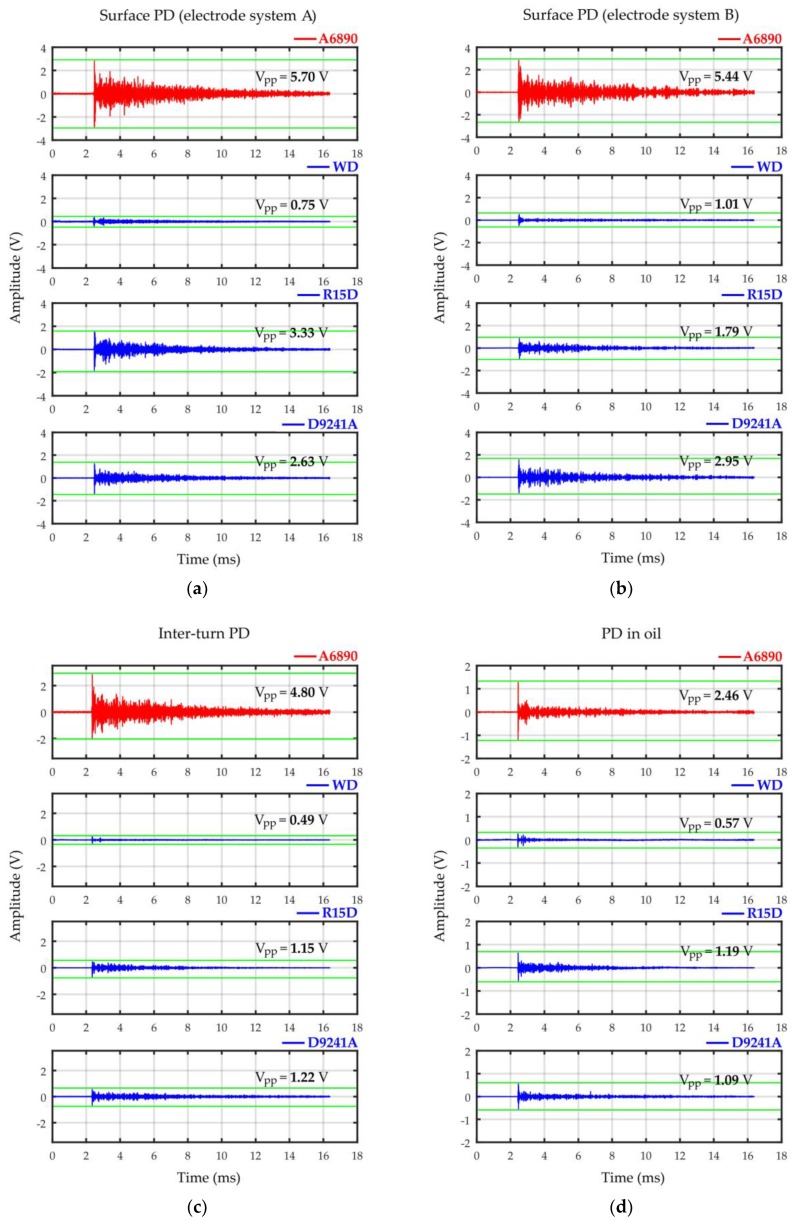
Comparison of typical AE waveforms registered for (**a**) surface PD generated in electrode system A, (**b**) surface PD generated in electrode system B, (**c**) interturn PD, and (**d**) PD in oil with the use of prototype (A6890) and commercial AE sensors (PAC WD, PAC R15D, and PAC D9241A).

The obtained results of the comparative analysis are presented graphically in Figure 23. In turn, in Figure 24 representative AE waveforms registered for the investigated PD forms are collected.

The analysis results prove that due to optimization of the transducer frequency response curve and application of two sensing elements, a very high detection sensitivity of partial discharges typical for power transformer insulation system was obtained. The highest differences in favor of prototype sensor were noted for interturn discharges. From the three commercial transducers, the low-frequency sensor type D9241A was characterized by the highest detection sensitivity of interturn discharges. Comparing to that sensor, the amplitude of the AE pulses registered with prototype sensor A6890 was ~3.9 higher (11.9 dB signal amplitude gain). The lowest difference was observed for surface discharges generated in the electrode system with a significant normal component of the electric field (electrode system B). In this case, the amplitude of the registered AE pulses was, on average, ~1.8 higher than for sensor D9241A and 2.8 times higher than for sensor R15D, which was respectively 5.2 dB and 9 dB signal amplitude gain.

**Figure 25:**
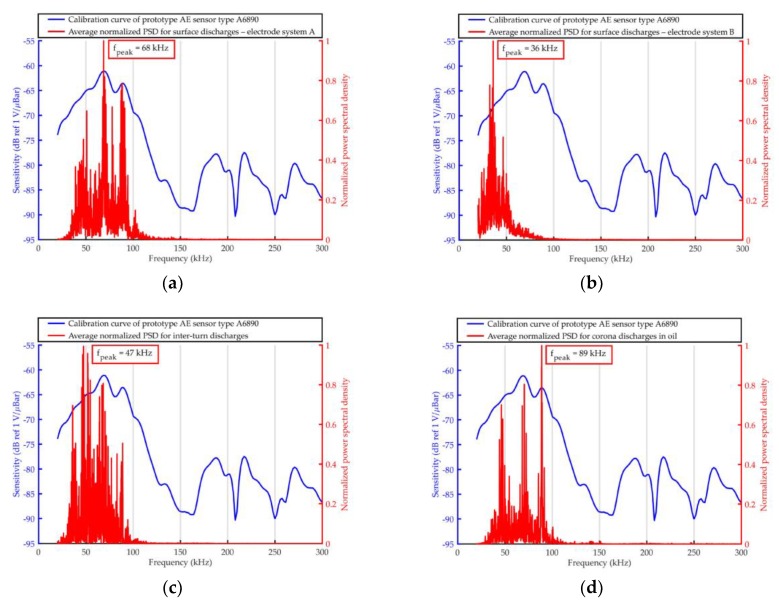
Average normalized PSD determined for AE signals emitted by (**a**) surface discharges generated in electrode system A, (**b**) surface discharges generated in electrode system B, (**c**) interturn discharges, and (**d**) partial discharges in oil, registered with the use of prototype AE sensor type A6890.

The frequency analysis of the AE pulses registered with prototype sensor A6890 (Figure 25) showed large similarity to the results obtained with broadband sensor type Olympus V101-RB. As expected, the highest similarity was noted for surface discharges generated in electrode system A with negligibly small normal component of the electric field. For both transducers, frequency peak value *f_peak_* in the spectrum was identical (68 kHz). In turn, for surface discharges generated in electrode system B with significant normal component of the electric field, the difference was equal to only 2 kHz (*f_peak_* = 38 kHz for Olympus V101-RB and *f_peak_* = 36 kHz for A6890). In the case of the remaining investigated PD types, the differences were slightly higher and equal 7 kHz for interturn discharges (*f_peak_* = 40 kHz for Olympus V101-RB and *f_peak_* = 47 kHz for A6890) and 9 kHz for partial discharges in oil (*f_peak_* = 98 kHz for Olympus V101-RB and *f_peak_* = 89 kHz for A6890).

## 8. Conclusions

A prototype AE sensor (named A6890) optimized for partial discharge detection in oil-filled power transformers was presented in the article. The main resonant frequencies of the A6890 sensor were selected based on extensive laboratory research, which allowed identification of acoustic frequencies emitted by partial discharges in oil–paper insulation. The measurement results showed that typical for power transformer forms of PDs generate AE signals, in which energy is transmitted in three main ranges: 20–45 kHz, 50–70 kHz, and 85–115 kHz, and dominant frequencies are equal to 40 kHz, 68 kHz, and 90 kHz, respectively. In the lowest band (20–45 kHz), noise associated with the deformation of magnetic domains in the transformer core and external acoustic disturbances may occur. Therefore, the prototype AE sensor was designed in order to operate in two remaining bands: 50–70 kHz and 85–115 kHz. For its manufacturing, two piezoelectric discs with opposite polarization directions and different heights were used. Fully differential design allowed to obtain the desired properties of the transducer, i.e., (1) two resonant frequencies for longitudinal acoustic waves (peaks at 68.7 kHz and 89 kHz), (2) high peak sensitivity (−61.1 dB ref. V/µbar), (3) low noise (removal of the common mode noise by the differential amplifier), and (4) wide frequency response (~30–100 kHz). The laboratory tests confirmed that the prototype transducer is characterized by ultrahigh sensitivity of partial discharge detection. Compared to popular commercial AE sensors, the average amplitude signal gain by a minimum 5.2 dB, and a maximum 19.8 dB was noted.

## Figures and Tables

**Table 1:** Parameters of the AE sensors used to determine the frequency of acoustic signals emitted by PD in oil–paper insulation.

Parameter	Sensor Type			
	Olympus V101-RB	PAC WD	PAC R15D	PAC D9241A
Operating frequency range, kHz	10–1000	100–1000	50–400	10–100
Peak sensitivity, dB (ref. V/µbar)	N/A	−64.25	−59.70	−69.2
Resonant frequency, kHz (ref. V/µbar)	550 ^1^	522	151.37	60.31
Output	single-ended	true differential ^2^	differential	differential
Category	Wideband nonresonant	wideband multiresonant	standard resonant	low-frequency

^1^ center frequency; ^2^ PAC WD has two sensing elements (piezoceramic disc and ring).

**Table 2:** The electrical parameters of the investigated types of partial discharges.

PD Type	Parameter				
	PD Inception Voltage U_i_ (kV)	PD Testing Voltage U_t_ (kV) ^1^	Apparent Charge q (pC)		
			Mean Value q_avg_	Maximum Value q_max_	Standard Deviation std(q)
Surface discharge (electrode system A)	19.6	20.3	410	597	112
Surface discharge (electrode system B)	12.9	14.1	1115	5430	155
Interturn discharge	18.5	19.9	792	3814	173
Partial discharge in oil	21.1	21.8	126	495	62

^1^ values of test voltage, at which the PD activity was stable (nonextinguishing) and AE pulses were registered.

**Table 3:** Frequency-domain parameters determined on the basis of the average PSD of AE pulses emitted by the investigated forms of partial discharges.

PD Type	AE Sensor	Frequency Domain Parameter		
		Peak Frequency (kHz)	Frequency Centroid (kHz)	Weighted Peak Frequency (kHz)
Surface discharge (electrode system A)	Olympus V101-RB	68.3	78.6	73.3
	PAC WD	96.3	117.2	106.2
	PAC R15D	153.3	142.3	147.7
	PAC D9241A	60.1	60.9	60.5
Surface discharge (electrode system B)	Olympus V101-RB	37.6	44.5	40.9
	PAC WD	28.4	48.2	37.0
	PAC R15D	46.4	83.2	62.1
	PAC D9241A	30.0	53.8	40.2
Interturn discharge	Olympus V101-RB	40.3	48.9	44.4
	PAC WD	30.9	72.3	47.3
	PAC R15D	46.6	100.2	68.4
	PAC D9241A	40.0	53.5	46.3
Partial discharge in oil	Olympus V101-RB	98.1	100.9	99.5
	PAC WD	105.8	167.7	133.2
	PAC R15D	115.2	127.5	121.2
	PAC D9241A	59.7	59.0	59.3

**Table 4:** Properties of the high-purity alumina used for matching layer.

Properties	Value	Unit
Main component	α-Al_2_O_3_	-
Purity	>99.5	wt-%
Density	3950	kg/m^3^
Open porosity	0	vol.-%
Average size of crystallites	10	µm
Compressive strength	3500	MPa
Young’s modulus	380	GPa
Poission’s ratio	0.22	-
Hardness	23	GPa
Acoustic velocity	9600	m/s
Acoustic impedance	37.9	MRayl

**Table 5:** Properties of the piezoelectric ceramic used.

Properties	Symbol	Value	Unit
Relative dielectric constant	*K^T^*	1900	-
Electromechanical coupling coefficients:			
Longitudinal coupling coefficient	*k_33_*	0.72	-
Transverse coupling coefficient	*k_31_*	0.36	-
Shear coupling coefficient	*k_15_*	0.68	-
Planar coupling coefficient	*k_p_*	0.63	-
Piezoelectric charge constants:			
Induced polarization in direction 3 (parallel to direction in which ceramic element is polarized) per unit stress applied in direction 3	*d_33_*	400 × 10^−12^	C/N
Induced polarization in direction 3 per unit stress applied in direction 1 (perpendicular to the direction in which ceramic element is polarized)	*d_31_*	175 × 10^−12^	C/N
Induced polarization in direction 1 per unit shear stress applied about a direction perpendicular to the direction in which ceramic element is polarized	*d_15_*	590 × 10^−12^	C/N
Dielectric dissipation factor (dielectric loss) ^1^	*tan σ*	≤2.00	%
Frequency constants:			
Frequency constant for thickness vibration mode	*N_T_*	2040	Hz·m
Frequency constant for planar vibration mode	*N_P_*	1980	Hz·m
Frequency constant for longitudinal vibration mode	*N_L_*	1500	Hz·m
Curie Point ^2^	*T_C_*	360	°C
Density	*ρ*	7600	kg/m^3^
Acoustic impedance	*Z*	31.5	MRayl

^1^ At 1 kHz, low field; ^2^ Maximum operating temperature = Curie point/2; ^3^ or m/V.
